# A*β* Peptide Originated from Platelets Promises New Strategy in Anti-Alzheimer's Drug Development

**DOI:** 10.1155/2017/3948360

**Published:** 2017-09-05

**Authors:** Mikhail Y. Inyushin, Priscila Sanabria, Legier Rojas, Yuriy Kucheryavykh, Lilia Kucheryavykh

**Affiliations:** ^1^School of Medicine, Department of Physiology, Universidad Central del Caribe, Bayamon, PR, USA; ^2^School of Medicine, Department of Biochemistry, Universidad Central del Caribe, Bayamon, PR, USA

## Abstract

The amyloid beta (A*β*) peptide and its deposits in the brain are known to be implicated in the neurodegeneration that occurs during Alzheimer's disease (AD). Recently, alternative theories views concerning both the source of this peptide and its functions have been developed. It has been shown that, as in all other known types of amyloidosis, the production of A*β* originates in blood cells or cells related to blood plasma, from which it can then spread from the blood to inside the brain, with the greatest concentration around brain blood vessels. In this review, we summarize research progress in this new area and outline some future perspectives. While it is still unclear whether the main source of A*β* deposits in AD is the blood, the possibility of blocking the chain of reactions that lead to constant A*β* release from the blood to the brain may be exploited in an attempt to reduce the amyloid burden in AD. Solving the problem of A*β* accumulation in this way may provide an alternative strategy for developing anti-AD drugs.

## 1. Introduction

It is known that Alzheimer's disease (AD) is the most common form of dementia, and it worsens over time. Current treatments cannot stop it from progressing, which emphasizes the desperate need for effective drugs. The hallmark of AD is pathological extracellular deposition of amyloid, which mainly consists of amyloid beta (A*β*) peptide, in the brain tissue of patients, and there is evidence supporting the concept that an imbalance between production and clearance of A*β* peptides is a very early, often initiating, factor in AD (reviewed in: [1]). The damage to the neurons and internal neuronal tangle formation is only a consequence of this imbalance, in which the action of soluble A*β* peptide oligomers plays a very important role [1–3]. While there has been a gradual accumulation of knowledge about the A*β* pathway, which is driving significant pharmaceutical industry drug research [4], one of the principal questions that might affect the therapeutic landscape is still unresolved: Where does A*β* peptide production originate?

## 2. A*β* Peptide Source

The prevailing hypothesis is that brain cells are responsible for A*β* peptide overproduction. It is known that both astrocytes [5, 6] and neurons [5, 7] produce and release monomeric A*β* to the extracellular space, which may aggregate and form fibrils if they become concentrated in the micromolar (*μ*M) range [8, 9]. It was shown that cultured human neurons with familiar AD-type mutations may, in only a few weeks, induce robust extracellular deposition of A*β*, including detergent-resistant plaques, in a human neural stem cell-derived three-dimensional (3D) culture system [10]. Neurons in this model have both amyloid precursor protein (APP) and presenilin 1 mutations and thus showed significant increases in the ratio of pathogenic A*β*42 to A*β*40 peptides compared with non-AD control neurons [2, 11]. A specialized gel was added to the culture to retain and accumulate the released A*β* [10], and this is a necessary element of the model; otherwise the aggregation is absent. Some gel components may be seeding points that start the A*β* plaque formation, because it was shown that peptides favor self-aggregation over cross-seeding with nonhomologous sequences [12]. The process of aggregation and amyloid fibril growth may be facilitated by seeding with preformed *β*-sheet amyloid resulting from cellular uptake, concentration, and aggregation of A*β* within endosomes/exosomes [13]. After *β*-sheet formation is initiated, this process resembles the spread of crystallization or prion-like propagation [14]. For example, addition of A*β* seeds and the external A*β* peptide can provoke aggregation in pure neuronal cultures that normally do not produce A*β* plaques without the gel. It was shown that while no A*β* deposition was observed in hippocampal neuronal cultures of postnatal APP transgenic (APP-tg) mice, A*β* deposition emerged when cultures were treated once with brain extract from aged APP-tg mice and the culture medium was continuously supplemented with additional synthetic A*β* [15]. These results confirmed that brain cells could supply enough A*β* to produce not only damaging oligomers but also the aggregated forms present in AD patients.

Recently, an alternative view has been developed. It was proposed that beta amyloidosis is developed from A*β* peptides (or from the A*β* seeds) that spread to the brain from the blood as in other common forms of amyloidosis, such as light-chain immunoglobulin amyloidosis, transthyretin amyloidosis, or reactive systemic amyloidosis. Can A*β* peptide come from blood? Previously, it was shown that platelets have relatively high concentrations of APP, which is mostly contained within alpha granules, and the alpha granule content is liberated upon platelet degranulation [16–20]. Practically all of the APP residing in blood plasma (~7 ng/ml) is thought to derive from platelets [16]. Platelets are also the primary source of A*β* peptide in human blood (~90%) [21], and this secreted peptide is similar to that found in the senile plaques of AD patients and is similarly increased in vivo by the presenilin 1 and presenilin 2 and APP mutations linked to familial AD [22]. The activated platelets in AD retain greater amounts of APP [23] while showing more platelet adhesion and thrombus formation [24]. In our recently published work we showed that A*β* peptide can be detected by immunocytochemistry in and around the blood vessels in the brain after experimental thrombosis and that this peptide is released from platelets [25]. We have also shown that during clot formation the density of platelets in the lumen of the thrombotic vessel is significantly increased (more than 300–500 times), thus allowing a massive release of A*β* peptide (directly or cleaved from released APP) at the site of clot formation [25]. It was known previously that microinfarcts are closely related to AD pathology [26–28], and there is a correlation with intracranial vessel arteriosclerosis [29, 30] in which microclots are chronically being formed in brain blood vessels during arteriosclerosis [31, 32].

It seems also that, compared with brain cells, in which APP is known to be bound to the cellular membrane during processing with successive secretases to produce the A*β* peptide, platelets use a somewhat distinct mechanism by releasing full-length APP. As shown by western blots and carboxyl-terminal and amino-terminal APP radioimmunoassay, activated platelets released a sedimentable full-length APP with the carboxyl-terminal epitope as well as a soluble APP lacking the carboxyl-terminal epitope (still containing A*β*, according to its molecular weight) [16]. During platelet degranulation some granules and dense bodies or their contents appear to be extruded into a serpentine system of lipid channels that is continuous with the platelet membrane [33]. It is also known that the material from alpha granules after the release from platelets forms a coating on the surface of the platelet [34]. Recently, specialized extracellular lipid vesicles (exosomes, microvessels, or microparticles) were found be emitted by platelets. These exosomes participate in the processing of many proteins (reviewed in [35]), but specifically it was shown that *β*-cleavage occurs in early endosomes, and the products are secreted from the cells in association with exosomes [36]. Interestingly, when platelets contact each other in the clot after aggregation, they change the A*β* production mechanism. It has been shown that platelets at high density secrete mainly A*β* ending at residue 40 (A*β*40) as a final product, while the A*β*42 level is not affected by cell density [37]. Alternatively, brain endothelial cell enzymes can cleave the platelet-released APP, forming A*β*, especially if activated platelets adhere directly to the endothelial cells [38].

To summarize, previous work has shown that platelets in blood vessels may (1) release full-length APP, (2) simultaneously process the released APP inside microvessels/exosomes (that have lipid membranes), (3) liberate A*β* as a final product, and (4) increase its specific production in case of being densely concentrated, as inside a thrombus. In addition, (5) APP may also be processed by the endothelial cells to generate A*β*.

Recently, the concept of very specialized membrane-bound *β*-/*γ*-secretases and the mechanism of APP processing were challenged, and it was suggested instead that there is a multiplicity of these proteins and mechanisms [39]. The available literature supports the possibility that APP processing to produce A*β* occurs upon cleavage in endosomes/exosomes, not in external membranes. It was shown that these microvesicles (microparticles) may contain enzymes of the cathepsin B family, a group of proteolytic enzymes whose cysteine or thiol groups are involved in the cleavage of substrates ([40], also in the book ed. [41]). The cysteine protease cathepsin B has been identified as a powerful *β*-secretase, and its inhibition reduces A*β* production from the precursor APP [42–44].

Alternatively, platelets have been shown to produce the initial seed of the aggregated A*β*, which can be spread to the brain [45]. It was shown that A*β* stimulates the integrin receptor in platelets, leading to the release of ADP and the protein clusterin, and this protein promoted *β*-sheet folding and the formation of fibrillar A*β* aggregates. The same authors have also shown that in transgenic AD model mice the antiplatelet agent clopidogrel (an ADP receptor [P2Y1, 2] blocker) reduced the amount of clusterin in the circulation and the incidence of cerebral amyloid angiopathy (CAA) [45]. In a transgenic mouse model of AD, platelets were found to be the major contributors to CAA, which then formed a shield of insoluble A*β* around brain blood vessels [46]. Interestingly, another antiplatelet drug, cilostazol, with a completely different mechanism of action that reduces platelet reactivity and degranulation, also reduced A*β*-induced cognitive deficits associated with A*β* accumulation [47–50], while aspirin, which reduces platelet aggregation but has no effect on degranulation, has no effect [51]. These results suggest that the degranulation of platelets is very important in AD development.

**Figure 1:**
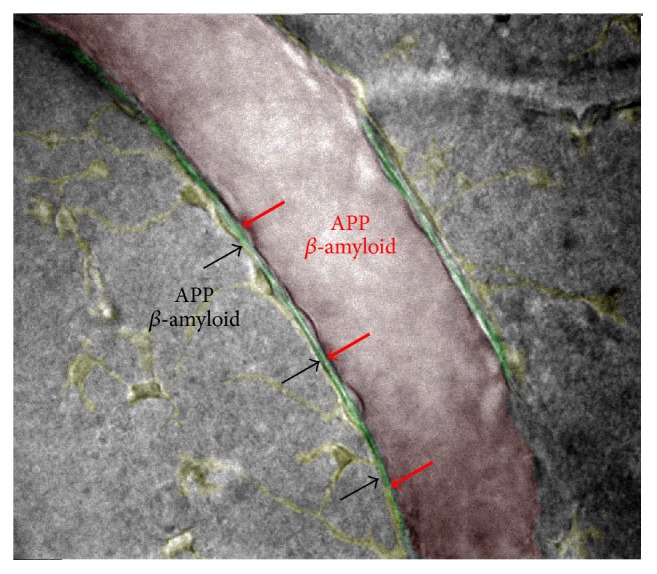
Beta-Amyloid from blood and from brain. Blood vessel lumens are shown in red, while perivascular space with basal lamina are shown in green on the sketch.

Production of A*β* in both brain and blood is beyond doubt. This fact led us to the hypothesis that both mechanisms of A*β* peptide production are important in the development of AD [25]. As a first step, there must be accumulation (aggregation) of platelets due to the response to some still-unknown septic or aseptic cause. According to this hypothesis, the A*β* peptide converted from APP and released from platelets (Figure 1, red arrows) spreads to the endothelium first and then to the perivascular space (Figure 1, green) and accumulates in the basal lamina. Another flow of A*β* peptide released from brain cells (Figure 1, black arrows) needs to pass the astrocyte barrier to reach the same perivascular space. Both brain- and blood-derived A*β* peptide may overwhelm the capacity of the existing clearance system.

## 3. The Perivascular Space Paradigm

Perivascular spaces are present around small as well as large blood vessels, in which case they are also known as Virchow-Robin spaces, forming the glymphatic perivascular system [52]. The main function of the glymphatic system is brain clearance: there is a constant flow along the perivenular and periarterial vascular spaces, from smaller to larger vessels [52, 53], and this flow increases during sleep [54]. The perivascular space extends only a few microns from small vessels (see Figure 1), but in bigger vessels it may extend a few millimeters. It is formed between the astrocytic end-feet enwrapping blood vessels (also known as the glia limitans, and the antibodies against aquaporin 4, the end-feet marker, can delimit its border) and the endothelium. In small vessels the perivascular space is known to be filled with basal lamina, a soft gel that consists of laminin, fibronectin, tenascin, collagens, and proteoglycans, which separates the astrocytic end-feet and endothelium cell layers and does not prevent the passage of macromolecules [55, 56]. In arterioles, the space between the end-foot and the endothelium includes also the smooth muscle cell layer. It is important to note that the accumulation of amyloid deposits follows a decreasing gradient from the inside of the vessel to the brain, not vice versa. It was shown that, during the development of AD angiopathy, amyloid accumulates initially in the perivascular space inside the basal lamina [57–59]. As the disease progresses, amyloid deposits extend into the smooth muscle cell layer in arterioles, leading to its loss. With end-stage disease, amyloid appears to spread to adjacent neuropil-forming dysphoric vessels [59] and produces complete disruption of the blood-brain barrier. Analyzing this data, it seems that amyloid moves from inside the blood vessel to the brain. Interestingly, amyloid deposits do not accumulate in the venous system. It may be important that elevated blood pressure in arteries drives the A*β* peptide from the lumen to the perivascular space, or alternatively it may be important that cerebral arterial pulsations drive the exchange of A*β* peptide in the perivascular space with fluids in the brain parenchyma [60]. In any case, the flow along the glymphatic system was shown to be very important for amyloid clearance [52, 53]. In a transgenic mouse model (APPswe/PS1dE9), it was shown that thrombotic cerebrovascular lesions induced a rapid transient increase in amyloid plaque burden and amyloid angiopathy in the area immediately surrounding the infarcted area [59]. The authors [61] suggested that this most probably happens due to interference with amyloid clearance pathways. On the other hand, we showed that thrombosis in wild-type mouse brains produces a massive release of APP from platelets, which is converted to A*β* [25]. These results suggest that both brain-derived and blood-derived A*β* peptide may be important and that platelets are the key players.

## 4. Not Only the Brain May Be Affected by A*β* Amyloid Deposits

### 4.1. Eye Retina and Crystal Lens

These deposits include retinal and lens amyloid accumulation, retinal nerve fiber layer loss, and retinal vascular changes [62].

### 4.2. Skeletal Muscle Myopathy

A*β*-containing structures as well as epitopes containing APP were described in some cases of inclusion-body myositis, the collection of diseases that are characterized by the vacuoles and filamentous inclusions in muscle fibers [63, 64]. Using electron microscopy, it was found that A*β*-immunoreactive structures were often in proximity to cytoplasmic tubulofilament inclusions (CTFs), but CTFs themselves never contained A*β* immunoreactivity [63]. Also, in some affected muscle cells A*β*-immunoreactive accumulations extended beyond the cell boundary, while in the majority of cases they were localized inside the cell (mainly near the sarcolemma boundary, but also deeply in the interior of the cell). Some A*β*-positive accumulations in the myocytes did not contain amyloid in *β*-pleated sheets, and this was hypothesized to be an early sign in the development of the myopathy [63]. Later, the fact that A*β*-immunoreactive accumulations can be found mainly inside the myocytes led many authors to the hypothesis that diseased muscle cells have the intrinsic genetic ability to produce augmented quantities of APP and A*β* during myositis (reviewed in [65]). In contrast, it was shown by the authors of the original discovery that inclusion-containing cells cultured from inclusion-body myositic muscle do not accumulate APP and can be restored to normality and easily innervated [66]. The same group also suggested that, during the course of the disease, the blood might produce A*β* and APP during inclusion-body myositis and all other inflammatory myopathies, as they found that human muscle macrophages express APP and have all the necessary machinery for its production.

### 4.3. Myocardium

While the heart may be affected by many diseases related to protein misfolding in myocardium tissue, new studies have shown that some cases of idiopathic cardiomyopathy are related to AD-associated presenilin gene variants [67]. Also, using modern imaging, proteomics, and echocardiographic methods, it was shown that patients with AD have diastolic dysfunction. A*β*40 and A*β*42 were present in the hearts of many patients with heart problems, but their expression was significantly increased in AD [68].

### 4.4. Skin

Amyloid protein deposit immunoreactivity was described many years ago in and around the endothelium of dermal blood vessels in aged AD patients and controls and was proposed as a marker of the disease in AD patients [69]. But another study found that skin accumulation of A*β* around blood vessels was unrelated to the severity of symptoms in AD patients, occurring also in some healthy subjects [70]. Interestingly, A*β* protein was also detected in the skin of patients with amyotrophic lateral sclerosis but not in controls [71]. In any case, it was very important that Joachim et al., 1989 [69], pointed out that the source of amyloid deposits in the skin and other organs was probably derived from a common circulating precursor, the blood. We have also shown that A*β* peptide can be found in and around skin blood vessels in mice after the experimental photothrombosis in the skin, as revealed by immunostaining, but not in normal skin [72]. These findings implicate thrombosis-related mechanisms as a probable source of this immunoreactivity.

A*β* in the skin may have implications for the development of vaccine against A*β* to cure AD, as patients with compromised clotting, like thrombophilia, thrombophlebitis, or similar diseases, could have additional inflammation problems. It is known that the development of the ACC-011AD vaccine (Elan-Wyeth Corp.) was halted due to patients developing skin lesions, which were suspected to be cases of blood vessel inflammation [73].

It is now clear that not only the brain but also other organs can be affected by A*β*, and the amount of deposition in these organs may be related or not to the severity of AD. As proposed by others [68], AD may be viewed either as a systemic disease or as a metastatic disorder, possibly leading to multiple organ failure.

## 5. Amyloid Deposits in AD Brain Contain Relatively Large Amounts of Other Proteins from Blood Plasma

Amyloid aggregates in AD may be of different types, with different solubilities in conventional solvents or detergents and different resistances to proteolysis. Plaques can be Congo Red-positive misfolded amyloid aggregates between the neurons, which typically have low solubility [4, 74–76], and there can be relatively soluble A*β* aggregates that typically appear as clusters of 10–20 nM ovoid structures [77]. The depositions in close proximity and around arteries (CAA) are also mainly insoluble. The major component of the AD deposits is the A*β* peptide, but there are also minor components that may affect solubility. One of the components, serum amyloid P component (AP), is a normal plasma constituent [78] produced by liver, and its concentration in blood plasma has been shown to be about 5-fold elevated in AD [79]. Besides its role in AD, AP is also a component of *β*-sheet-containing plaques in other types of amyloidosis, such as light-chain immunoglobulin amyloidosis or reactive systemic amyloidosis. AP reduces solubility and also prevents proteolysis of amyloid deposits, but since it is not itself an enzyme inhibitor, it is protective only when bound to the deposits [80]. This implies that blood components participate in the formation of many brain amyloid aggregates in AD. Why then is it not possible that the A*β* peptide is itself (at least partially) derived from blood?

We need to mention here that some other minor components of AD deposits definitely come from the brain: it was shown that the synaptic vesicle protein synuclein 1 or its components are visible in AD plaques as well [81].

## 6. Why Are Platelets Activated in the AD Brain?

The systemic view of AD may be explained by systemic release of A*β*, but in any case the most severe symptoms are developed in the brain. Why is the brain the target of A*β* release in AD if there is no traumatic thrombosis? And why are only some parts of the brain affected?

It is well known that the different parts of the brain are not affected equally, at least according to the density and distribution of senile plaques and neurofibrillary tangles. During development, the disease primarily affects the entorhinal cortex [82–84], which is a major input and output structure of the hippocampal formation (affected in the next stage of the disease) but is also known to convey olfactory information [85, 86]. It was shown that the olfactory bulb is also definitely affected and may be affected even before the cortex [87, 88]. It was proposed that the olfactory tracts might provide a portal for entry into the brain of any putative pathogenic agent(s) that might be responsible for the induction of senile plaques and/or neurofibrillary tangles [88]. This led to the old theory of an infectious etiology for AD and the association with different brain viruses, bacteria, or prions (reviewed in [89, 90]). The evidence for human transmission of A*β* pathology and CAA after the treatment of children with human cadaver-derived growth hormone was reported, and it was suggested that this was an example of the prion-like spread of AD [91].

Recently, another explanation has been proposed. It was shown that A*β* release may be a specific defense against septic agents. If such agents are transmitted to the brain, the body responds by releasing A*β* peptide to kill them. If this defense mechanism is somehow compromised, it has been suggested that this may lead to overproduction of the A*β* peptide.

## 7. The Antimicrobial Effect and A*β* Peptide Oligomers

The antimicrobial effect of the A*β* peptide is clearly established. Its activity against different viruses has been shown [92–95] as well as its strong antibiotic activity against both Gram-negative and Gram-positive bacteria and yeast [95–97]. This allows one to suggest that A*β* oligomers are a hitherto-unrecognized antimicrobial agent that functions as a normal component of the innate immune system [95]. Two different mechanisms have been proposed to explain this A*β* function: (i) the trapping of bacteria and (ii) pore-forming antibiotic activity.

(i) It was shown that the development of A*β* protofibrils can prevent pathogen adhesion to host cells, thus explaining the protective effect of A*β* against* Salmonella* bacterial infection [97]. It was shown that* Salmonella* bacterial infection of the brains of transgenic 5XFAD mice resulted in rapid seeding and accelerated A*β* deposition, which closely colocalized with the invading bacteria. This evidence led the authors to suggest that any infectious or sterile inflammatory stimuli could drive amyloidosis [97].

(ii) It has been shown that A*β* peptide oligomers perforate cell membranes and form (at low concentrations) tetrameric channels permeable to K^+^ ions. At higher concentrations they form giant multilevel Ca^++^-permeable pores, which are mainly hexamers [98–100]. An excess of Ca^++^ permeability through these pores leads to cell death, as it induces unrestricted calcium flux into the cell, leading to dyshomeostasis [99].

There is a similarity of the A*β* peptide oligomer pore-forming effect with that of other peptide antibiotics (e.g., cecropin A or nystatin) that kill target cells by formation of large pores in the membrane [101–103]. This led us to hypothesize that A*β* peptide released from platelets or cleaved from platelet-released APP works effectively as a natural antibiotic during clot formation following injury or platelet degranulation in response to other septic or aseptic (inflammatory) stimuli [25].

## 8. What Is a New and Alternative Strategy to Fighting AD?

While the A*β* hypothesis is driving the majority of current research on AD, the development of effective drugs against the disease has stalled. These drugs are directed against A*β*, its precursor APP, or the *β*- and *γ*-secretase enzymes from the known A*β* pathway. Knowledge about this pathway is driving significant pharmaceutical industry drug research [4], and while current drugs help mask the symptoms of AD, they do not treat the underlying disease or delay its progression. None of the A*β*‐targeted phase 3 clinical trials in AD has shown a statistically significant benefit in achieving its prespecified clinical endpoints [1]. Several of these trials against A*β* and *γ*-secretases had to be halted because of side effects that may not have been target related [104, 105].

Analysis of the literature suggests a new approach that takes into account not only brain-derived but also blood-derived A*β* and proposes that platelets are the key players. We further suggest an alternative strategy in which AD is treated through the reduction (inhibition) of pathologically excessive release of A*β* (through APP) by platelets. A breakthrough AD drug must treat the underlying disease and stop or delay the cell damage that eventually leads to the worsening of symptoms.

## 9. Platelets and Their Receptors That Induce A*β* Release Are Involved in AD

**Figure 2:**
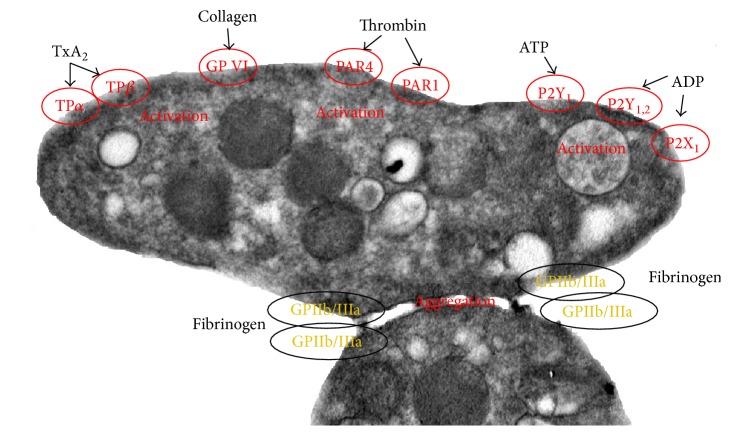
Activation of different types of receptors induces degranulation of platelets.

It is known that normal or pathological platelet activation may take place after attachment and adhesion events or as the effect of many other activation factors, such as thromboxane A2, the ADP/ATP ratio, thrombin, platelet-activating factor (PAF), collagen, and many others. The activation pathways are usually mediated by specific receptors, some of which are shown in Figure 2. Platelet-activation signaling pathways have been known for many years, and over time it has become clear that there is a complex and sophisticated signaling and amplification network (reviewed in [106]). The specific pathways that are most important in the pathological activation related to AD are not known, and this needs to be studied.

In Figure 2, we show a few of the many receptors that activate platelets and their possible relation to AD.

*Thromboxane A2 (TxA2) Prostanoid G Protein-Coupled Receptors with Two Alternatively Spliced Isoforms (α and β).* TxA2 receptor antagonists have been shown to block iPF2*α*III-induced increases in A*β* secretion [107, 108].

*Glycoprotein VI (GPVI) Collagen Receptor.* Honokiol (extracted from magnolia), a specific antagonist of collagen receptor glycoprotein VI, which is expressed on human platelets [109], was shown to attenuate A*β*-induced memory impairment, which was attributed to its antioxidant activity [110].

*Proteinase-Activated Receptors (PARs).* These members of the G protein-coupled receptor family are usually activated by thrombin in platelets. Activation of PARs may result in AD pathogenesis (reviewed by [111]).

*Purinergic ATP-ADP G Protein-Coupled Receptors (P2X, P2Y).* It was shown that clopidogrel (a P2Y1, 2 ADP receptor blocker) significantly reduced the incidence of CAA [45]. Using selective purinergic receptor agonists and antagonists in vitro and in AD animal models it was shown that these receptors represent novel therapeutic targets for the treatment of AD [112].

*Glycoprotein IIb/IIIa Receptors.* The progression of dementia in AD patients with fast cognitive decline was shown to be correlated with the levels of activated glycoprotein IIb-IIIa complex and P-selectin [113].

 It is especially interesting that the ADP and TxA2 receptors both signal via the specific G protein Gq. Temporal elimination of Gq greatly inhibits functioning of both these receptors. It was shown that agonists (activating ligands) of* retinoid X nuclear hormone receptors* (RXRs) bind directly to Gq and very effectively inhibit platelet aggregation, due to dysfunction of the ADP and TxA2 receptors, but only weakly affect functioning of collagen (GP VI) receptors [114]. This is definitely a nongenomic effect because the lack of a nucleus in these cells [114]. On the other hand, it was shown that oral administration of the RXR agonist bexarotene to a mouse model of AD resulted in enhanced clearance of soluble A*β* within hours. The A*β* intraneuronal amyloid deposit area was reduced more than 50% within just 72 hours. Furthermore, bexarotene stimulated the rapid reversal of cognitive, social, and olfactory deficits and improved neural circuit function [115, 116].

## 10. Conclusions

Analyzing the literature, we agree with Schmaier [117] that results concerning APP and A*β* peptide structure and function suggest that AD is in part a thrombohemorrhagic disorder and conclude that platelets and their activation mechanisms are most probably involved. Moreover, these insights give us hope for an alternative strategy in anti-AD drug development focused on newly discovered blood-related pathways of A*β* release and aggregation.
